# Five‐Year Real‐World Safety of Inotuzumab Ozogamicin Before Hematopoietic Stem Cell Transplantation in B‐Cell Precursor Acute Lymphoblastic Leukemia

**DOI:** 10.1002/ajh.27637

**Published:** 2025-02-24

**Authors:** Marcos de Lima, Partow Kebriaei, Francesco Lanza, Christina Cho, Gizelle Popradi, Manmeet Kaur, Mei‐Jie Zhang, Fan Zhang, Richa Shah, Erik Vandendries, Kofi Asomaning, Stephanie Dorman, Matthias Stelljes, David I. Marks, Wael Saber

**Affiliations:** ^1^ Blood and Marrow and Cellular Therapy Program Ohio State University Columbus Ohio USA; ^2^ MD Anderson Cancer Center Houston Texas USA; ^3^ Ospedale di Ravenna, Ravenna University of Bologna Bologna Italy; ^4^ John Theurer Cancer Center Hackensack University Medical Center Hackensack New Jersey USA; ^5^ McGill University Health Centre Montreal Quebec Canada; ^6^ Center for International Blood and Marrow Transplant Research Medical College of Wisconsin Milwaukee Wisconsin USA; ^7^ Pfizer Inc Shanghai China; ^8^ Pfizer Inc New York New York USA; ^9^ Pfizer Inc Cambridge Massachusetts USA; ^10^ Pfizer Inc New York New York USA; ^11^ University Hospital Münster Germany; ^12^ University Hospitals Bristol NHS Trust Bristol UK

**Keywords:** ALL, marrow/stem cell transplantation—clinical results in acute leukemia, stem cell transplantation, veno‐occlusive disease, VOD

To the Editor,

Acute lymphoblastic leukemia (ALL) is rapidly fatal without appropriate treatment [1]. Chemotherapy and targeted agents have improved survival rates, but some patients need allogeneic hematopoietic stem cell transplantation (HSCT) or chimeric antigen T cell therapy to achieve long‐term remission [1]. Inotuzumab ozogamicin (InO) is a CD22‐directed antibody‐drug conjugate approved for relapsed/refractory (R/R) B‐cell precursor ALL based on results from the phase 3 INO‐VATE trial [2]. Despite its substantial clinical benefit, InO has been linked to an increased risk of hepatic veno‐occlusive disease/sinusoidal obstruction syndrome (VOD/SOS) [3]. VOD/SOS can occur from injury to the sinusoidal endothelium of the liver, typically following myeloablative (high‐dose) chemo‐radio therapy and HSCT, and can evolve into severe liver dysfunction and multi‐organ failure [4]. Pooled results from INO‐VATE and the phase 1/2 Study 1010 demonstrated higher transplant‐related mortality (TRM) in patients with R/R ALL receiving InO before HSCT versus those receiving standard therapy before HSCT, due in part to complications from VOD/SOS [5].

This observational, non‐interventional, post‐authorization safety study used real‐world data from the Center for International Blood and Marrow Transplant Research (CIBMTR) to evaluate post‐HSCT outcomes in patients with B‐cell precursor ALL who received InO and subsequent HSCT in the US between August 18, 2017, and August 17, 2022. The CIBMTR is a research collaboration between the National Marrow Donor Program/Be The Match and the Medical College of Wisconsin charged with collecting data on allogeneic HSCTs performed in the US (https://cibmtr.org). The National Marrow Donor Program/Be The Match Central Institutional Review Board, which is fully accredited by the Association for the Accreditation of Human Research Protection Programs, reviewed and approved this study. The study was conducted in accordance with the FDA Guidance for Industry and FDA Staff: Best Practices for Conducting and Reporting of Pharmacoepidemiologic Safety Studies Using Electronic Healthcare Data Sets, Good Pharmacoepidemiology Practices issued by the International Society for Pharmacoepidemiology, the International Society for Pharmacoeconomics and Outcomes Research guidance, and Pharmaceutical Research and Manufacturers Association guidelines.

The primary focus of this descriptive analysis was adults with ALL (and the subset of adults with R/R ALL) receiving first allogeneic HSCT after InO; outcomes in adults receiving allogeneic HSCT before InO are briefly described. Post‐HSCT outcomes included TRM, non‐TRM, relapse, overall survival (OS), and post‐HSCT adverse events (AEs) of interest, including VOD/SOS. Multivariable analyses examined prognostic factors for TRM at 18 months and VOD/SOS at 100 days. See Supporting Information for additional details.

In all, 5891 patients with ALL received allogeneic HSCT during the 5‐year study period and were reported to the CIBMTR; 3377 were excluded because they had not consented to research or were from embargoed or non‐participating centers (Figure S1). Of the remaining 2514 patients, 261 were adults who received ≥ 1 dose of InO and proceeded to first allogeneic HSCT (Table S1); 244 (156 with R/R ALL) had sufficient follow‐up data and were included in this analysis.

Median (range) age of the 244 patients was 39 years (18–75) and 141 (58%) were male (Table S2); 117 (48%) received InO as monotherapy, and 109 (45%) received InO in combination with ≥ 1 other agent. After InO treatment, 198 (81%) patients achieved complete response (CR)/CR with incomplete hematologic recovery, and 161/215 (75%) evaluable patients achieved measurable residual disease negativity. Median (range) time from last InO dose to HSCT was 2.4 months (0.6–26.2). Disease status at HSCT was CR1 in 87 (36%) patients, CR2 in 114 (47%) patients, and CR ≥ 3 in 26 (11%) patients. Liver toxicity prophylaxis consisted of ursodiol alone (*n* = 198 [81%]), ursodiol and defibrotide (*n* = 20 [8%]), ursodiol and other non‐specified agents (*n* = 6 [2%]), or defibrotide alone (*n* = 2 [1%]); 14 (6%) patients received no liver toxicity prophylaxis, and 4 (2%) had no available data.

Median (range) post‐HSCT follow‐up in patients with ALL and R/R ALL was 10.6 (0.4–54.1) and 8.8 (0.4–50.8) months, respectively (Table 1). In all, 119 (49%) patients with ALL and 66 (42%) patients with R/R ALL spent < 30 days inpatient in the first 100 days after HSCT. Following HSCT, 18‐month OS (95% CI) in patients with ALL and R/R ALL, respectively, was 54% (47–61) and 50% (41–58) (Figure S2); 18‐month TRM (95% CI) was 22% (17–27) and 25% (18–32) (Figure S3). The primary investigator‐assessed causes of death were VOD/SOS (*n* = 13 [26%]), graft‐versus‐host disease (GVHD; *n* = 11 [22%]), organ failure (*n* = 10 [20%]), hemorrhage (*n* = 3 [6%]), interstitial pneumonitis (*n* = 4 [8%]), infection (*n* = 5 [10%]), septic shock (*n* = 2 [4%]), graft failure (*n* = 1 [2%]), and other (*n* = 1 [2%]). The most common causes of TRM in patients with R/R ALL were VOD/SOS (*n* = 9 [24%]), GVHD (*n* = 7 [19%]), and organ failure (*n* = 7 [19%]).

**TABLE 1:** Post‐HSCT outcomes.

Outcome	Patients with ALL (*N* = 244)	Patients with R/R ALL^a^ (*N* = 156)
Follow‐up duration, median (range), months	10.6 (0.4–54.1)	8.8 (0.4–50.8)
Overall survival, % (95% CI)		
12 months	61 (54–67)	56 (47–64)
18 months	54 (47–61)	50 (41–58)
Transplant‐related mortality, % (95% CI)		
6 months	17 (13–22)	20 (14–27)
12 months	20 (15–25)	23 (17–30)
18 months	22 (17–27)	25 (18–32)
Non‐transplant‐related mortality, % (95% CI)		
12 months	19 (14–25)	21 (15–28)
18 months	24 (19–31)	25 (18–33)
Relapse, % (95% CI)		
12 months	33 (27–39)	34 (26–42)
18 months	40 (33–47)	40 (32–48)
Continued CR,^b^ *n* (%)	226 (93)	138 (88)
VOD/SOS incidence within 100 days, % (95% CI)	14 (10–19)	18 (12–24)
VOD/SOS mortality,^c^ %	36	40
Time from HSCT to VOD/SOS, median (range), months	0.5 (0.2–3.0)	0.4 (0.2–2.6)
Engraftment syndrome within 100 days, *n* (%)	18 (7)	13 (8)
Time from HSCT to engraftment syndrome, median (range), days	14 (2–42)	14 (2–42)
Total inpatient days in first 100 days post HSCT, *n* (%)		
< 30	119 (49)	66 (42)
30–59	60 (25)	46 (29)
60–100	31 (13)	21 (13)
Not reported	34 (14)	23 (15)

Abbreviations: ALL, acute lymphoblastic leukemia; CR, complete remission; HSCT, hematopoietic stem cell transplantation; R/R, relapsed/refractory; VOD/SOS, veno‐occlusive disease/sinusoidal obstruction syndrome.

^a^ Includes patients who did not undergo HSCT during CR1 with any line of therapy.

^b^ Continued CR is defined as a patient who received HSCT during CR and CR was sustained post‐HSCT, where CR is defined as < 5% blasts in bone marrow, no blasts with Auer rods, no extramedullary disease, and no disease progression for at least 4 weeks.

^c^ Cumulative incidence estimate at 18 months following post‐HSCT VOD/SOS.

AEs occurring in ≥ 30% of patients with ALL within 100 days after HSCT were bacterial infection (*n* = 125 [51%]), viral infection (*n* = 107 [44%]), and acute grades II–IV GVHD (*n* = 105 [43%]) (Table S3). Rates were similar among patients with R/R ALL.

Thirty‐five (14%) patients with ALL and 27 (17%) with R/R ALL developed VOD/SOS within 100 days after HSCT. Cumulative incidence of VOD/SOS at 100 days in the respective groups was 14% and 18% (Table 1; Figure S4). Of 35 patients who developed VOD/SOS, 15 had mild and 20 had severe VOD/SOS. A dual‐alkylating conditioning regimen was used in 2 (13%) mild cases and 5 (25%) severe cases of VOD/SOS; myeloablative conditioning was used in 12 (80%) mild cases and 13 (65%) severe cases of VOD/SOS. Eight of 35 patients with VOD/SOS received defibrotide as liver toxicity prophylaxis. Median (range) time from HSCT to VOD/SOS was 15 days (6–91) in patients with ALL and 12 days (6–79) in patients with R/R ALL. Thirteen patients received defibrotide treatment either alone (*n* = 3) or with other agents (*n* = 10). Twenty‐two patients with VOD/SOS within 100 days died within 18 months post HSCT, 14 with VOD/SOS as a cause of death. Post‐HSCT VOD/SOS mortality was 36% in patients with ALL and 40% in patients with R/R ALL. Cumulative incidence (95% CI) of VOD/SOS at 100 days in patients with time from last InO dose to HSCT of < 1, 1.1–1.6, 1.7–3, and > 3 months was not estimable, 25% (14–38), 12% (5–21), and 13% (7–20), respectively. See Tables S4 and S5 for additional VOD/SOS data.

In multivariable analyses (*n* = 204), Karnofsky performance status score < 90 and dual‐alkylating conditioning were negative prognostic factors for 18‐month TRM (Table S6). Dual‐alkylating conditioning was the only negative prognostic factor for VOD/SOS within 100 days. Cumulative InO exposure and the combination of an alkylating agent with total body irradiation were not associated with increased VOD/SOS within 100 days. Median (range) cumulative InO dose was 2.2 mg/m^2^ (0.3–6.9).

Forty‐three adults with ALL had allogeneic HSCT before InO and went on to second or greater HSCT; 12‐month TRM was 27% and the 12‐month relapse rate was 8%. Eight (19%) patients developed VOD/SOS after second or greater HSCT; cumulative incidence of VOD/SOS at 100 days was 21%. Post‐HSCT VOD/SOS mortality was 75%. Outcomes in patients with R/R ALL (*n* = 41) were similar but with a lower 12‐month relapse rate (3%).

In this 5‐year real‐world study of patients with B‐cell precursor ALL who received InO prior to HSCT, including heavily pretreated patients, the InO safety profile was similar to that previously observed [2, 5]. The cumulative incidence of VOD/SOS in patients with R/R ALL (18%) was similar to that observed in the pooled analysis of the phase 1/2 Study 1010 and INO‐VATE trials (19%) [5]. TRM was lower than previously reported. In INO‐VATE, TRM (95% CI) was 37% (26–47) at 12 months and 38% (27–49) at 18 months (Pfizer data on file). In the pooled analysis, TRM (95% CI) was 38% (28–47) at 12 months and 39% (30–49) at 24 months [5]. The lower TRM observed here may relate to a number of factors including increased experience with InO, better use of VOD/SOS mitigation strategies, and changes in transplant GVHD prophylaxis. Our multivariable analysis highlights the adverse impact of dual‐alkylating conditioning regimens on the development of post‐HSCT VOD/SOS and TRM, corroborating previous findings [3, 5]. We suggest avoiding dual alkylators when possible and using alternative conditioning agents. Patients may also benefit from enrolling in clinical trials testing novel prophylactic treatment for post‐HSCT endothelial dysfunction syndromes.

When making decisions regarding InO treatment, potential risk factors for the development of VOD/SOS must be carefully considered. Precautions such as avoiding hepatotoxic conditioning regimens and close monitoring of patients should be implemented to minimize risk. Modifiable risk factors for VOD/SOS have been published by the European Society for Blood and Marrow Transplantation, including high‐dose myeloablative conditioning regimens, oral or high‐dose busulfan, high‐dose treosulfan, and high‐dose total body irradiation‐based regimens [6]. Use of human leukocyte antigen‐mismatched and unrelated HSCT donors may also be modifiable risk factors. Additionally, agents used for GVHD prophylaxis, such as sirolimus + methotrexate + tacrolimus and methotrexate + cyclosporin/tacrolimus, can contribute to VOD/SOS. Prophylactic ursodeoxycholic acid is recommended and should be administered from initiation of conditioning, or sooner, until day 90+ after HSCT [7]. The current data suggest time from last InO dose to HSCT and number of InO cycles do not impact VOD/SOS risk, which likely reflects the practice of limiting the number of cycles to two (80% of all patients).

## Author Contributions

M.J.Z., E.V., K.A., D.I.M., and W.S. contributed to the study design/conception. P.K., M.K., M.J.Z., and W.S. contributed to the acquisition of the data. All authors were involved in the analysis and interpretation of data, contributed to revising manuscript drafts, and reviewed and approved the final manuscript.

## Consent

Patients provided consent for their data to be used for research.

## Conflicts of Interest

M.d.L.: consulting or advisory role: Amgen, Celgene, Incyte, and Pfizer; research funding: Celgene and Pfizer. P.K.: consulting or advisory role: Jazz, Kite, and Pfizer. F.L.: consulting or advisory role: AbbVie, Alexion, and Amgen; research funding: Pfizer. C.C.: none. G.P.: consulting or advisory role, and honoraria: AbbVie, Gilead, Kite, Kyowa Kirin, Merck, Novartis, Pfizer, Seattle Genetics, Servier, and Taiho; consulting or advisory role: Daiichi Sankyo and Mallinckrodt; speakers' bureau participant: AbbVie, Gilead, Jazz, Merck, Novartis, PeerVoice, Pfizer, Seattle Genetics, and Servier; research funding: Jazz and Novartis. M.K., M.J.Z., and W.S.: none. F.Z., R.S., E.V., K.A., and S.D.: employment: Pfizer. R.S., E.V., K.A., and S.D.: stock and stock ownership interests: Pfizer. M.S.: honoraria, consultant or advisory role, and research funding: Pfizer. D.M.: consulting, educational activities: Pfizer, Kite, and Novartis.

## Supporting information


**Data S1.** Supporting Information.

## Data Availability

Upon request, and subject to review, Pfizer will provide the data that support the findings of this study. Subject to certain criteria, conditions and exceptions, Pfizer may also provide access to the related individual de‐identified participant data. See https://www.pfizer.com/science/clinical‐trials/trial‐data‐and‐results for more information.

## References

[1] T. Terwilliger and M. Abdul‐Hay , “Acute Lymphoblastic Leukemia: A Comprehensive Review and 2017 Update,” Blood Cancer Journal 7 (2017): e577, 10.1038/bcj.2017.53. 28665419 PMC5520400

[2] H. M. Kantarjian , D. J. DeAngelo , M. Stelljes , et al., “Inotuzumab Ozogamicin Versus Standard Therapy for Acute Lymphoblastic Leukemia,” New England Journal of Medicine 375 (2016): 740–753, 10.1056/NEJMoa1509277. 27292104 PMC5594743

[3] H. M. Kantarjian , D. J. DeAngelo , A. S. Advani , et al., “Hepatic Adverse Event Profile of Inotuzumab Ozogamicin in Adult Patients With Relapsed or Refractory Acute Lymphoblastic Leukaemia: Results From the Open‐Label, Randomised, Phase 3 INO‐VATE Study,” Lancet Haematology 4 (2017): e387‐e398, 10.1016/S2352-3026(17)30103-5. 28687420

[4] F. Bonifazi , F. Barbato , F. Ravaioli , et al., “Diagnosis and Treatment of VOD/SOS After Allogeneic Hematopoietic Stem Cell Transplantation,” Frontiers in Immunology 11 (2020): 489, 10.3389/fimmu.2020.00489. 32318059 PMC7147118

[5] D. I. Marks , P. Kebriaei , M. Stelljes , et al., “Outcomes of Allogeneic Stem Cell Transplantation After Inotuzumab Ozogamicin Treatment for Relapsed or Refractory Acute Lymphoblastic Leukemia,” Biology of Blood and Marrow Transplantation 25 (2019): 1720–1729, 10.1016/j.bbmt.2019.04.020. 31039409

[6] M. Mohty , F. Malard , A. S. Alaskar , et al., “Diagnosis and Severity Criteria for Sinusoidal Obstruction Syndrome/Veno‐Occlusive Disease in Adult Patients: A Refined Classification From the European Society for Blood and Marrow Transplantation (EBMT),” Bone Marrow Transplantation 58 (2023): 749–754, 10.1038/s41409-023-01992-8. 37095231

[7] P. Kebriaei , C. Cutler , M. de Lima , et al., “Management of Adverse Events Associated Wtih Inotuzumab Ozogamicin: Expert Panel Review,” Bone Marrow Transplant 53 (2018): 449–456, 10.1038/s41409-017-0019-y. 29330398 PMC5897380

