# Supplementary material for: Five‐Year Real‐World Safety of Inotuzumab Ozogamicin Before Hematopoietic Stem Cell Transplantation in B‐Cell Precursor Acute Lymphoblastic Leukemia
Source: Am J Hematol. 2025 Feb 24;100(5):909–12. doi: 10.1002/ajh.27637 (PMC11966346; doi:10.1002/ajh.27637)
Supplement: Supplementary file 1 — Data S1. Supporting Information. [file AJH-100-909-s001.pdf]

SUPPLEMENTARY METHODS

Eligibility criteria

Patients with B-cell precursor ALL who were treated with at least one dose of InO (at any timepoint and as any line of therapy), received subsequent HSCT at a participating center during the 5-year period following the US FDA approval of InO (August 18, 2017, to August 17, 2022), and whose data were reported to the CIBMTR at the Comprehensive Report Form level were included. Additionally, patients with transplant prior to InO use were included.

The Comprehensive Report Form level of data collection captures detailed patient-, disease-, and transplant-related data in addition to standard data collection. Data are collected pre-HSCT, 100 days after HSCT, 6 months after HSCT, annually until 6 years after HSCT, and biannually thereafter until death or loss to follow-up. Patients were excluded if they were treated at a transplant center that was not participating in the study or was embargoed from contributing to research studies because the center did not meet CIBMTR data quality standards, or if the patient did not give consent to participate in the CIBMTR research database.

Post-HSCT outcomes

TRM was defined as the time from HSCT to death within 28 days of HSCT or death from any cause without prior relapse/progression after HSCT. NTRM was defined as the time from HSCT to death after 28 days post HSCT from any cause with prior relapse/progression post HSCT. Post-transplant relapse was defined as the time from HSCT to first relapse after HSCT, either without death after relapse or with death after 28 days post HSCT. Post-transplant OS was defined as the time from HSCT to death due to any cause. Post-HSCT follow-up was the time from HSCT to date of last contact. For these outcomes, data were censored at the date the patient was last known

to be alive in the absence of confirmation of death (or relapse for post-transplant relapse). The duration (in months) of these outcomes was calculated as follows: [date of event/competing event/last known to be alive − date of transplant + 1]/30.4375.

Statistical methods

The study baseline timepoint was the date of the current HSCT. All analyses were based on descriptive statistics. Unadjusted *P* values are provided, but no definite conclusions were made based on *P* values, and no adjustments for multiple comparisons were applied. Categorical variables are summarized using counts and percentages. Continuous variables are summarized using descriptive statistics (median, minimum, maximum and number of patients).

Post-HSCT OS was summarized using the Kaplan-Meier method; median event times were summarized with 95% confidence intervals (CI) calculated using the method described by Brookmeyer and Crowley.[1] TRM, NTRM, post-transplant relapse, and day 100 VOD/SOS incidence were summarized using competing-risks analyses. Competing events were post-transplant relapse for TRM, TRM for NTRM and post-transplant relapse, and death without VOD/SOS for VOD/SOS incidence. The cumulative incidence of events was summarized with 95% CIs calculated on the basis of on the cumulative incidence function using the SAS macro by Lin et al,[2] which is based on the method described by Kalbfleisch et al.[3] For time-to-event endpoints, patients who had not yet experienced the event of interest by the end of follow-up were censored.

Missing event dates (except for death dates) were handled as follows: if the day of the month was missing for any date used in a calculation, the first of the month was used to replace the missing date unless the calculation resulted in a negative time duration (i.e., date of onset

cannot be prior to day one date). In this case, the date resulting in zero time duration was used. If the day of the month and the month was missing for any date used in a calculation, January 1 was used to replace the missing data. Missing death dates were handled as follows: if the entire date was missing, it was not imputed, and the time-to-event was censored at the date that the patient was last known to be alive; if the day of the month was missing, the maximum of the full (non-imputed) day after the date of last contact and the first of the month was used to replace the missing date; if the day of the month and the month were both missing, the maximum of the full (non-imputed) day after the date of last contact and January 1 was used to replace the missing date.

The Clark Completeness Index was used to quantify the effect of losses to follow-up in patients whose centers submitted follow-up information.[4] The Completeness Index was calculated as the ratio of observed time (entry time into study until study end or event [death]) to potential time. Follow-up was determined at last contact date on 1-month, 100-day, 6-month, and 12-month follow-up forms submitted by transplant centers. Clark Completeness Index Scores for the 244 adult patients who had follow-up data reported were calculated as 100% at 1 month and 100 days, 98% at 6 months, 93% at 12 months and 88% at 18 months.

Multivariable analyses examined prognostic factors for TRM at 18 months and VOD/SOS at 100 days. For TRM, a Fine and Gray's sub-distribution hazards model was used to account for the competing risk event (post-HSCT relapse). Logistic regression was used to evaluate VOD/SOS within 100 days post HSCT. The following nine variables were used in the multivariable analyses: patient age (18–29 vs 30–59 vs ≥ 60), patient sex (male vs female), Sorror HCT-CI (0 vs 1–2 vs ≥ 3), Karnofsky performance score prior to transplant (90–100 vs 10–80), sirolimus use as GVHD prophylaxis given (yes vs no), disease status prior to HSCT

(CR1 vs CR2 vs advanced), conditioning regimen intensity and dual alkylators (myeloablative conditioning [MAC]/dual alkylators vs MAC/no dual alkylators vs reduced-intensity conditioning/non-MAC), donor type (human leukocyte antigen-identical sibling vs other related vs unrelated), and number of cycles of InO treatment prior to HSCT.

The data lock for this report, when data collection forms were last evaluated, was August 17, 2022

References

1. Brookmeyer R, Crowley J. A confidence interval for median survival time. Biometrics. 1982;38(1):29-41.
2. Lin G, So Y, Johnston G. Analyzing survival data with competing risks using SAS® software. SAS Global Forum 2012 [Internet]. 2012. Available from: https://support.sas.com/resources/papers/proceedings12/344-2012.pdf.
3. Kalbfleisch J, Prentice R. The statistical analysis of failure time data. New York: Wiley;1980.
4. Clark TG, Altman DG, De Stavola BL. Quantification of the completeness of follow-up. Lancet. 2002;359:1309-10.

Supplementary Table S1. Demographics and baseline characteristics of all adults with ALL who received InO prior to first HSCT.

	**Patients with ALL (N = 261)**	**Patients with R/R ALL (N = 166)**
Male, *n* (%)	152 (58)	86 (52)
Age, median (range), y	39 (18–75)	36 (18–75)
Sorror HCT-CI score, *n* (%)		
0	36 (14)	23 (14)
1–2	97 (37)	57 (34)
≥3	121 (46)	80 (48)
Not reported	7 (3)	6 (4)
Lines of therapy prior to InO, *n* (%)		
No treatment given	28 (11)	6 (4)
First line	71 (27)	26 (16)
Salvage 1	47 (18)	39 (23)
Salvage 2	44 (17)	39 (23)
Salvage >2	42 (16)	33 (20)
Not reported/outlier[a]	29 (11)	23 (14)
Median (range)	2 (0–8)	3 (0–8)
Cycles of InO, *n* (%)		
1	83 (32)	54 (32)
2	122 (47)	77 (46)
3	37 (14)	24 (15)
4	13 (5)	7 (4)
5	1 (<1)	1 (1)
Not reported	5 (2)	3 (2)
InO administration, *n* (%)		
Single agent	120 (46)	81 (49)
Combined with other chemotherapy/systemic therapy	112 (43)	62 (37)
Not reported/outliers	29 (11)	23 (14)
Response to InO, *n* (%)		
CR	159 (61)	87 (52)
CRi[b]	50 (19)	40 (24)

No CR	47 (18)	36 (22)
Not reported	5 (2)	3 (2)
MRD-negativity rate after InO, *n/N* (%)	167/227 (74)	NA
Time from last InO dose to HSCT, median (range), mo	2.4 (0.6–26.2) (*n* = 247)	2.3 (0.6–26.2)
Disease status prior to HSCT, *n* (%)		
CR1	94 (36)	-
CR2	121 (46)	121 (73)
CR ≥3	28 (11)	28 (17)
1st relapse	10 (4)	10 (6)
≥3rd relapse	3 (1)	3 (2)
Primary induction failure	4 (2)	4 (2)
Not reported	1 (<1)	0 (0)
Lines of therapy prior to HSCT, *n* (%)		
First line	8 (3)	0 (0)
Salvage 1	57 (22)	19 (11)
Salvage 2	59 (23)	39 (23)
Salvage >2	114 (44)	89 (54)
Not reported/outliers[a]	23 (9)	19 (11)
Median (range)	3 (1–10)	4 (2–10)
Abnormal AST level prior to HSCT, n (%)	81 (31)	53 (32)
AST level prior to HSCT, median (range), units/L	0.8 (0.3–3.6) (*n* = 252)	0.9 (0.3–3.3) (*n* = 160)
Abnormal total serum bilirubin prior to HSCT, *n* (%)	19 (7)	17 (10)
Total serum bilirubin prior to HSCT, median (range), mg/dL	0.4 (0–4.4) (*n* = 251)	0.4 (0.2–4.4) (*n* = 159)
Conditioning regimen intensity, *n* (%)		
Myeloablative	135 (52)	88 (53)
Reduced intensity/non-myeloablative	113 (43)	70 (42)
Unknown/not reported	13 (5)	8 (5)
Busulfan used in conditioning regimen, *n* (%)	30 (11)	20 (12)
Thiopeta used in conditioning regimen, *n* (%)	20 (8)	16 (10)
Donor type, n (%)		
HLA-identical sibling	70 (27)	42 (25)

Other related	67 (26)	50 (30)
Unrelated	124 (48)	74 (45)

Abbreviations: ABT, antibody-based therapy; ALL, acute lymphoblastic leukemia; AST, aspartate transaminase; CR, complete remission; CR1/CR2/CR3, first/second/third complete remission; HCT-CI, Hematopoietic Cell Transplantation Comorbidity Index; HLA, human leukocyte antigen; HSCT, hematopoietic stem cell transplantation; InO, inotuzumab ozogamicin; MRD, measurable residual disease; NA, not available; R/R, relapsed/refractory; TBI, total body irradiation.

[a] Inconsistency between disease status and total lines of therapy.

Supplementary Table S2. Demographics and baseline characteristics of adults with ALL who received InO prior to first HSCT and had follow-up data.

Characteristic	Patients with ALL (*N* = 244)	Patients with R/R ALL (*N* = 156)
Male, *n* (%)	141 (58)	81 (52)
Age, median (range), y	39 (18–75)	36 (18–75)
Sorror HCT-CI score, *n* (%)		
0	35 (14)	23 (15)
1–2	87 (36)	52 (33)
≥3	116 (48)	76 (49)
Not reported	6 (2)	5 (3)
ALL cytogenetic risk group, *n* (%)		
Ph+ ALL	42 (17)	NA
B-cell precursor ALL with iAMP21	3 (1)	NA
11q23 and t(4;11)	15 (6)	NA
Favorable B-cell precursor ALL: high hyperdiploidy	7 (3)	NA
Intermediate B-cell precursor ALL: normal karyotype, all other abnormalities not associated with a risk group	160 (66)	NA
Adverse B-cell ALL: monosomy 7, complex karyotype, del(7q), t(8;14), t(11;19), del(11q), tetraploidy	17 (7)	NA
Lines of therapy prior to InO, *n* (%)		
No treatment given	27 (11)	6 (4)
First line	69 (28)	26 (17)
Salvage 1	45 (18)	37 (24)
Salvage 2	43 (18)	38 (24)
Salvage >2	42 (17)	33 (21)
Not reported/outlier[a]	18 (7)	16 (10)
Median (range)	2 (0–8)	3 (0–8)
Cycles of InO, *n* (%)		
1	79 (32)	NA
2	115 (47)	NA
≥ 3	49 (20)	NA
Not reported	1 (<1)	NA
InO administration, *n* (%)		
Single agent	117 (48)	NA

Combined with other chemotherapy/systemic therapy	109 (45)	NA
InO + ABT	3 (3)	NA
InO + ABT + other	2 (2)	NA
InO + TKI	9 (8)	NA
InO + chemo	33 (30)	NA
InO + chemo + ABT	41 (38)	NA
InO + chemo + ABT + TKI	3 (3)	NA
InO + chemo + ABT + others	1 (1)	NA
InO + chemo + TKI	9 (8)	NA
InO + chemo + other	4 (4)	NA
InO + other	4 (4)	NA
Not reported/outliers	18 (7)	NA
Time from last InO dose to HSCT, median (range), months	2.4 (0.6–26.2) (*n* = 235)	NA
Disease status prior to HSCT, *n* (%)		
CR1	87 (36)	-
CR2	114 (47)	114 (73)
CR ≥3	26 (11)	26 (17)
1st relapse	10 (4)	10 (6)
≥3rd relapse	3 (1)	3 (2)
Primary induction failure	3 (1)	3 (2)
Not reported	1 (<1)	0 (0)
Lines of therapy prior to HSCT, *n* (%)		
First line	8 (3)	0 (0)
Salvage 1	54 (22)	19 (12)
Salvage 2	57 (23)	37 (24)
Salvage >2	111 (45)	87 (56)
Not reported/outlier[a]	14 (6)	13 (8)
Median (range)	3 (1–10)	4 (2–10)
Abnormal AST prior to HSCT, *n* (%)	77 (32)	50 (32)
AST level prior to HSCT, median (range), units/L	0.8 (0.3–3.6) (*n* = 242)	0.9 (0.3–3.3) (*n* = 154)
Abnormal ALT prior to HSCT, *n* (%)	29 (12)	NA
ALT level prior to HSCT, median (range), units/L	0.6 (0–2.2) (*n* = 122)	NA
Abnormal total serum bilirubin prior to HSCT, *n* (%)	19 (8)	17 (11)

Total serum bilirubin prior to HSCT, median (range), mg/dL	0.4 (0–4.4) (*n* = 241)	0.4 (0.2–4.4) (*n* = 153)
Conditioning regimen intensity, *n* (%)		
Myeloablative	127 (52)	83 (53)
Reduced intensity/non-myeloablative	111 (45)	69 (44)
Not reported	6 (2)	4 (3)
TBI used in conditioning regimen, *n* (%)	155 (64)	NA
≤8 Gy	61 (39)	NA
>8 Gy	94 (61)	NA
Busulfan used in conditioning regimen, *n* (%)	29 (12)	20 (13)
<9 mg/kg IV	15 (52)	NA
≥9 mg/kg IV	14 (48)	NA
Donor type, *n* (%)		
HLA-identical sibling	64 (26)	40 (26)
Other related	64 (26)	47 (30)
Matched unrelated (8/8)	82 (34)	NA
Partially matched unrelated (7/8)	14 (6)	NA
Mismatched unrelated (≤6/8)	3 (1)	NA
Multi-donor	17 (7)	NA
Unrelated[b]	-	69 (44)

Abbreviations: ABT, antibody-based therapy; ALL, acute lymphoblastic leukemia; ALT, alanine transaminase; AST, aspartate transaminase; chemo, chemotherapy; CR, complete remission; CR1/CR2/CR3, first/second/third complete remission; HCT-CI, Hematopoietic Cell Transplantation Comorbidity Index; HLA, human leukocyte antigen; HSCT, hematopoietic stem cell transplantation; InO, inotuzumab ozogamicin; IV, intravenous; MRD, measurable residual disease; NA, not available; R/R, relapsed/refractory; TBI, total body irradiation; TKI, tyrosine kinase inhibitor.

Some data were not available in the subset of patients with R/R ALL.

[a] Inconsistency between disease status and total lines of therapy.

[b] Further details of unrelated donors not available in the R/R ALL subset.

Supplementary Table S3. Adverse events of interest occurring within 100 days after HSCT in patients with follow-up data.

**Adverse event within 100 days, *n* (%)**	**Patients with ALL (*N* = 244)**	**Patients with R/R ALL (*N* = 156)**
Viral infection	107 (44)	69 (44)
Bacterial infection	125 (51)	88 (56)
Maximum grade acute GVHD[a]		
I	26 (11)	16 (10)
II	72 (30)	43 (28)
III	17 (7)	11 (7)
IV	16 (7)	10 (6)

Abbreviations: ALL, acute lymphoblastic leukemia; GVHD, graft-versus-host disease; HSCT, hematopoietic stem cell transplantation; R/R, relapsed/refractory.

Adverse events occurring in ≥30% of patients shown.

[a]Acute GVHD grading follows the Consensus criteria (Przepiorka D, Weisdorf D, Martin P, et al. [1995] 1994 Consensus Conference on Acute GVHD Grading. Bone Marrow Transplant 15:825–828.) Acute GVHD was evaluated up to 100 days after HSCT.

Supplementary Table S4. Additional data for patients with ALL who developed VOD/SOS and had follow-up data.

	**Patients with ALL**
VOD/SOS by lines of therapy prior to HSCT, *n/N* (%)	
Firstline	1/8 (13)
Salvage 1	3/54 (6)
Salvage 2	11/57 (19)
Salvage >2	18/111 (16)
VOD/SOS by number of InO cycles, % (95% CI)	
1 cycle (n = 79)[a]	17 (9–26)
2 cycles (n = 115)[a]	11 (6–18)
≥3 cycles (n = 49)	18 (9–30)
VOD/SOS by post-HSCT cyclophosphamide use, % (95 % CI)	
Yes (n = 97)[a]	16 (9–24)
No (n = 147)[a]	14 (9–20)
VOD/SOS by busulfan dose, *n/N* (%)	
<9 mg/kg IV	2/15 (13)
≥9 mg/kg IV	3/14 (21)
VOD/SOS by TBI dose, % (95% CI)	
≤8 Gy (n = 61)[a]	15 (7–25)
>8 Gy (n = 94)	18 (11–27)
VOD/SOS by InO with other agents across all lines of therapies prior to HSCT, % (95% CI)	
InO + chemo only (n = 47)	15 (6–27)
InO + chemo + ABT only (n = 76)	12 (6–20)
InO + chemo + ABT ± TKI/others (n = 50)	20 (10–32)
InO + chemo ± TKI/others (n = 37)[a]	14 (5–27)
No chemo (n = 34)[a]	12 (3–26)

Abbreviations: ABT, antibody-based therapy; ALL, acute lymphoblastic leukemia; chemo, chemotherapy; CI, confidence interval; HSCT, hematopoietic stem cell transplantation; InO, inotuzumab ozogamicin; TBI, total body irradiation; TKI, tyrosine kinase inhibitor; VOD/SOS, veno-occlusive disease/sinusoidal obstruction syndrome.

[a] VOD/SOS information not reported for one patient.

Supplementary Table S5. Details of patients who developed VOD/SOS.

ID	Age	Race	Sex	Sorror HCT-CI score	No. therapy lines prior to HSCT	Karnofsky score	Disease status prior to HSCT	Time from last dose of InO to HSCT (mo)	GVHD prophylaxis	Donor
1	44.7	White	Male	3–4	3	10–80	CR2	3.4	Cy ± others	Unrelated
2	33.2	White	Male	1–2	≥5	90–100	CR2	4.1	Cy ± others	Other related
3	33.2	White	Male	3–4	3	10–80	CR2	3.0	Tac + MTX ± others (not Cy, MMF)	Unrelated
4	40.9	White	Male	3–4	4	10–80	CR2	2.0	Tac + MTX ± others (not Cy, MMF)	HLA-identical sibling
5	28.3	NR	Male	1–2	≥5	10–80	CR2	1.4	Tac + MMF ± others (not Cy)	Unrelated
6	48.2	White	Male	3–4	≥5	10–80	CR2	3.3	Tac + MTX ± others (not Cy, MMF)	Unrelated
7	66.4	White	Female	3–4	≥5	10–80	CR2	3.3	Cy ± others	Unrelated
8	23.5	NR	Female	0	NR	90–100	CR2	1.5	CsA + MTX ± others (not Cy, Tac, MMF)	HLA-identical sibling
9	20.9	White	Male	0	≥5	90–100	CR3+	1.6	CsA + MTX ± others (not Cy, Tac, MMF)	HLA-identical sibling
10	20.1	White	Female	3–4	≥5	10–80	CR3+	22.7	Tac + MTX ± others (not Cy, MMF)	Unrelated
11	42.1	White	Female	1–2	3	10–80	CR2	2.2	Tac + MTX ± others (not Cy, MMF)	HLA-identical sibling
12	23.0	White	Female	1–2	≥5	90–100	CR3+	6.0	CsA + MTX ± others (not Cy, Tac, MMF)	HLA-identical sibling
13	37.8	White	Male	3–4	2	10–80	CR1	1.2	Tac + MTX ± others (not Cy, MMF)	Unrelated
14	39.8	White	Male	5	4	10–80	REL1	2.3	Tac + MMF ± others (not Cy)	Unrelated
15	64.0	White	Male	1–2	4	NR	CR3+	2.1	Tac + MTX ± others (not Cy, MMF)	HLA-identical sibling

ID	Age	Race	Sex	Sorror HCT-CI score	No. therapy lines prior to HSCT	Karnofsky score	Disease status prior to HSCT	Time from last dose of InO to HSCT (mo)	GVHD prophylaxis	Donor
16	43.2	Black or African American	Female	3–4	3	90–100	CR2	1.4	Cy ± others	Other related
17	50.7	White	Female	3–4	3	10–80	CR2	4.7	Tac + MTX ± others (not Cy, MMF)	Unrelated
18	23.9	White	Male	3-4	≥5	90–100	CR2	1.6	Cy ± others	Other related
19	27.3	White	Male	3–4	3	NR	CR3+	5.1	Cy ± others	Other related
20	46.1	White	Male	1–2	4	10–80	CR1	1.1	Cy ± others	Other related
21	21.6	White	Female	1–2	2	90–100	CR2	1.2	CsA + MMF ± others (not Cy, Tac)	Other related
22	22.1	Asian	Male	0	3	90–100	CR2	1.5	Cy ± others	Other related
23	20.0	White	Male	0	≥5	90–100	CR1	0.8	Tac + MTX ± others (not Cy, MMF)	Unrelated
24	62.3	White	Female	7	3	90–100	CR2	1.6	Cy ± others	Other related
25	48.9	White	Male	1–2	3	90–100	CR3+	2.1	Cy ± others	Other related
26	25.0	White	Male	3–4	3	10–80	CR1	1.0	Cy ± others	Other related
27	26.5	White	Male	0	2	90–100	CR2	1.4	Cy ± others	Unrelated
28	54.2	White	Male	1–2	≥5	10–80	CR1	0.7	Tac + MMF ± others (not Cy)	Other related
29	36.9	American Indian or Alaska Native	Female	1–2	≥5	10–80	CR1	1.9	Cy ± others	Unrelated
30	19.0	NR	Male	3–4	3	NR	CR1	4.0	Cy ± others	Other related
31	21.0	White	Female	1–2	4	90–100	CR2	4.3	Tac + MTX ± others (not Cy, MMF)	Unrelated

ID	Age	Race	Sex	Sorror HCT-CI score	No. therapy lines prior to HSCT	Karnofsky score	Disease status prior to HSCT	Time from last dose of InO to HSCT (mo)	GVHD prophylaxis	Donor
32	20.7	White	Male	1–2	≥5	10–80	CR2	5.4	Tac + MMF ± others (not Cy)	Unrelated
33	24.7	White	Male	1–2	4	10–80	CR2	1.7	Tac + MTX ± others (not Cy, MMF)	HLA-identical sibling
34	73.4	White	Male	0	1	90–100	CR1	1.5	Cy ± others	Unrelated
35	32.9	White	Male	1–2	NR	90–100	CR2	2.3	Tac + MTX ± others (not Cy, MMF)	HLA-identical sibling

Abbreviations: CR1/CR2/CR3, first/second/third complete remission; Cy, cyclophosphamide; GVHD, graft-versus-host disease; HCT-CI, Hematopoietic Cell Transplantation Comorbidity Index; HLA, human leukocyte antigen; HSCT, hematopoietic stem cell transplantation; InO, inotuzumab ozogamicin; MMF, mycophenolate mofetil; MTX, methotrexate; NR, not reported; REL1, first relapse; Tac, tacrolimus.

Supplementary Table S6. Multivariable analyses of TRM at 18 months and VOD/SOS within 100 days.

Covariate	Number evaluable	HR/OR (95% CI)	*P* value
**TRM at 18 months**			
Karnofsky score			<0.0001[a]
<90 vs 90–100	81 vs 111	3.00 (1.56–5.79)	0.0010
Unknown vs 90–100	9 vs 111	7.55 (2.88–19.76)	<0.0001
Conditioning regimen intensity and dual alkylators			0.0002[a]
MAC/no dual alkylators vs MAC/dual alkylators	97 vs 14	0.15 (0.06–0.37)	<0.0001
RIC/non-MAC vs MAC dual alkylators	90 vs 14	0.26 (0.11–0.62)	0.0027
RIC/non-MAC vs MAC/no dual alkylators	90 vs 97	1.73 (0.90–3.32)	0.0972
**VOD/SOS within 100 days**			
Conditioning regimen intensity and dual alkylators			0.0131[a]
MAC/no dual alkylators vs MAC/dual alkylators	97 vs 14	0.26 (0.08–0.86)	0.0275
RIC/non-MAC vs MAC dual alkylators	89 vs 14	0.15 (0.04–0.53)	0.0032
RIC/non-MAC vs MAC/No dual alkylators	89 vs 97	0.57 (0.24–0.36)	0.2064

Abbreviations: MAC, myeloablative conditioning; RIC, reduced-intensity conditioning; TRM, transplant-related mortality; VOD/SOS, veno-occlusive disease/sinusoidal obstruction syndrome.

[a] Wald test with 2 degrees of freedom.

Supplementary Figure S1. Patient flow chart

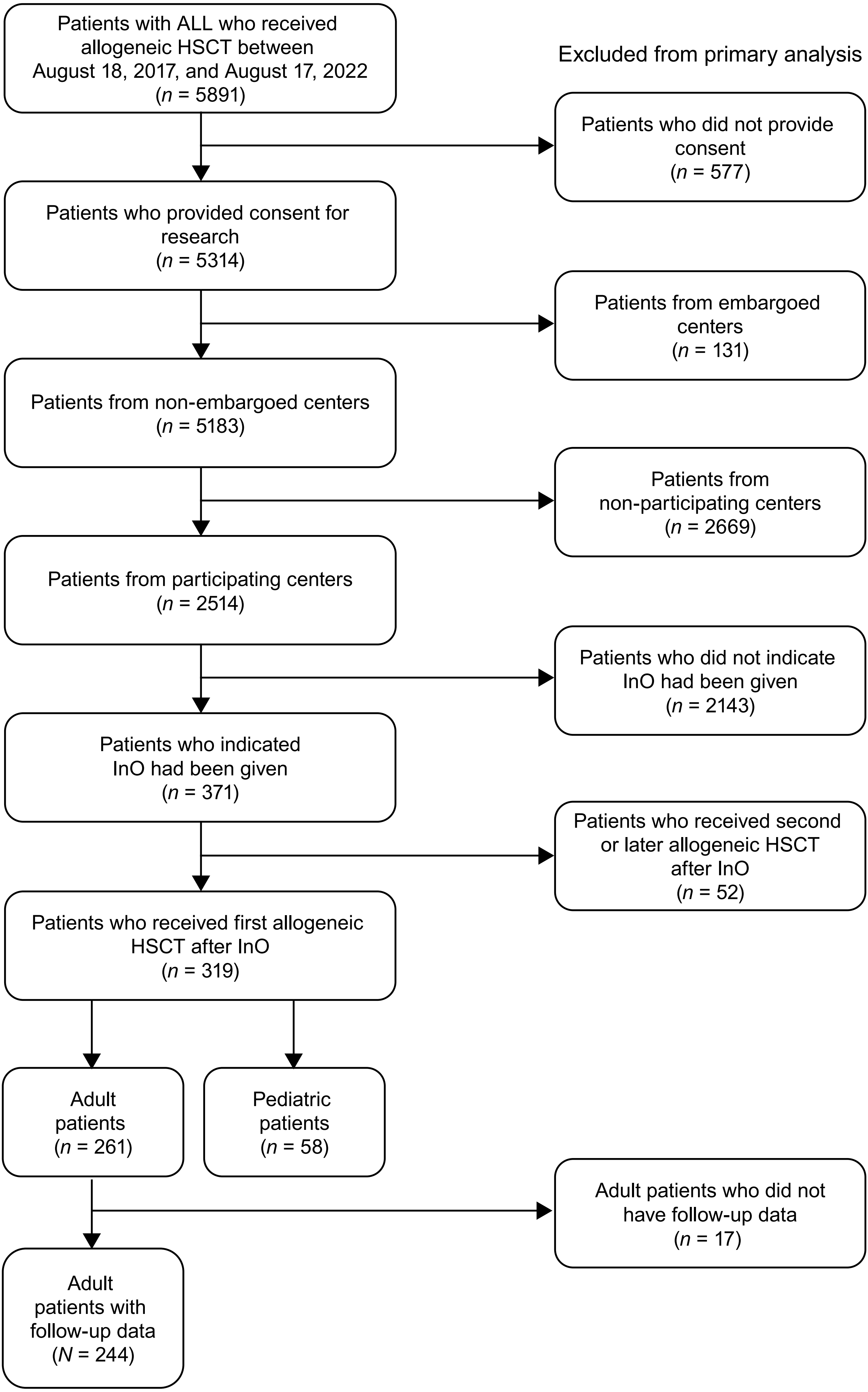

Abbreviations: ALL, acute lymphoblastic leukemia; HSCT, hematopoietic stem cell transplantation; InO, inotuzumab ozogamicin.

Supplementary Figure S2. Overall survival within 18 months post HSCT

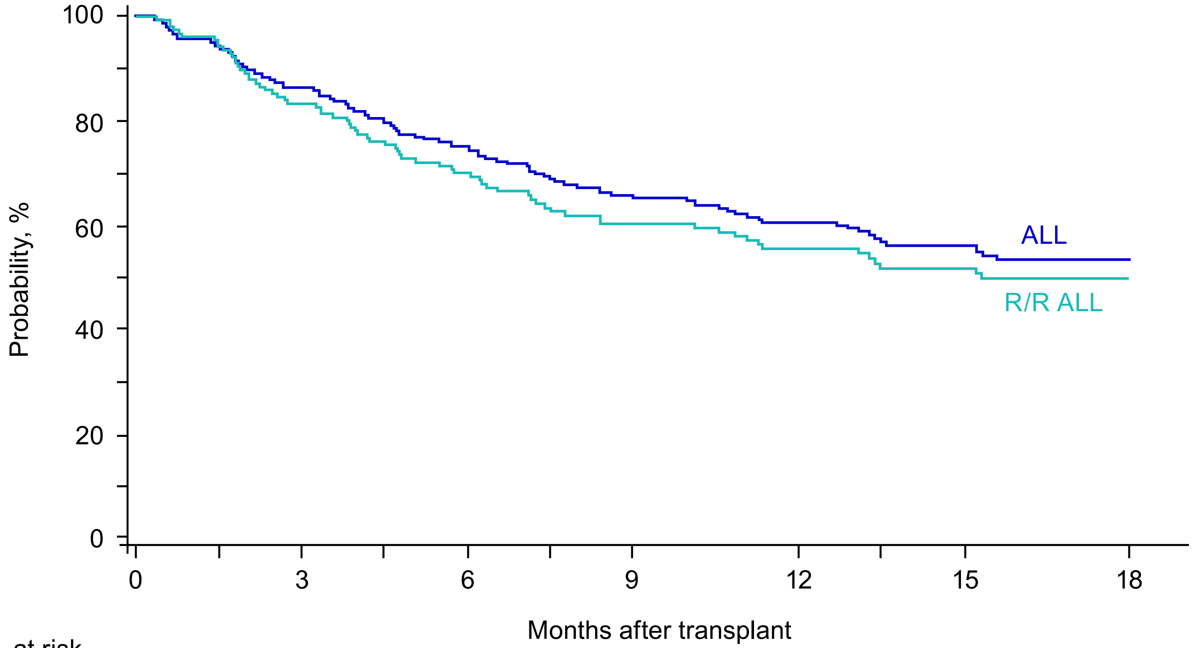

No. at risk

	0	3	6	9	12	15	18
ALL	244	210	167	131	116	85	75
R/R ALL	156	130	101	78	71	54	48

Abbreviations: ALL, acute lymphoblastic leukemia; HSCT, hematopoietic stem cell transplantation; R/R, relapsed/refractory.

Supplementary Figure S3. Transplant-related mortality within 18 months post HSCT.

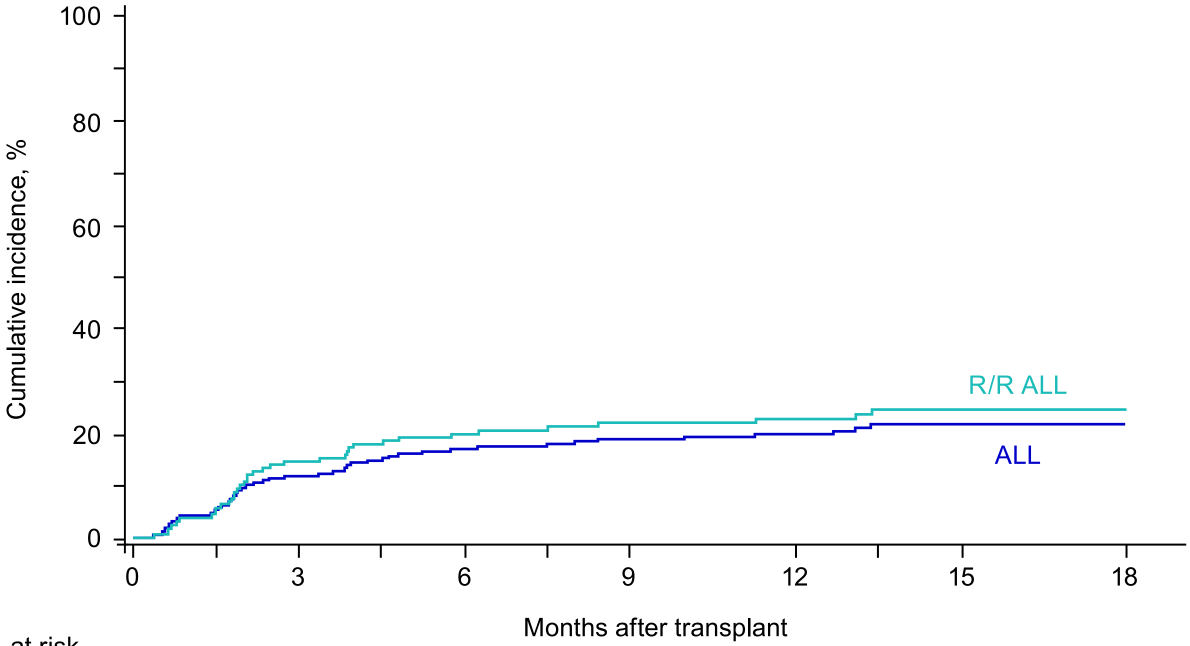

No. at risk

ALL	244	191	135	109	92	62	57
R/R ALL	156	117	83	66	57	40	37

Abbreviations: ALL, acute lymphoblastic leukemia; HSCT, hematopoietic stem cell transplantation; R/R, relapsed/refractory.

Supplementary Figure S4. Cumulative incidence of VOD/SOS within 100 days post HSCT.

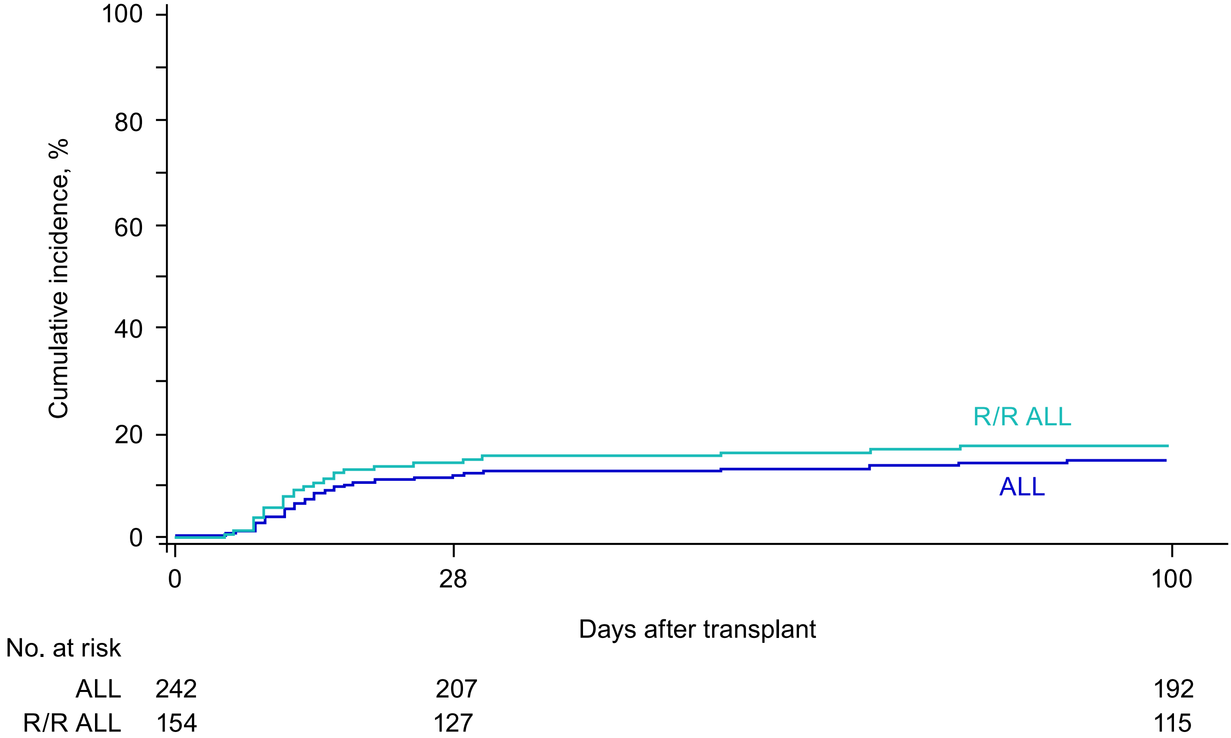

Abbreviations: ALL, acute lymphoblastic leukemia; HSCT, hematopoietic stem cell transplantation; R/R, relapsed/refractory; VOD/SOS, veno-occlusive disease/sinusoidal obstruction syndrome.

Two patients did not have the applicable post-HSCT VOD/SOS data and were excluded from the figure.
